# Changes in intestinal microbiota in patients with pancreatic cancer: a systematic review and meta-analysis

**DOI:** 10.3389/fmicb.2025.1619323

**Published:** 2025-09-01

**Authors:** Meng Liang, Zheng Liu, Rui Zhang, Ji-Hong Yang, Xiao-Wei Wang, Nan Zhang

**Affiliations:** ^1^Department of Hepatobiliary Surgery, Affiliated Hospital of Hebei University, Baoding, China; ^2^Department of Gastroenterology, Affiliated Hospital of Hebei University, Baoding, China; ^3^Department of Cardiology, Affiliated Hospital of Hebei University, Baoding, China; ^4^Department of Critical Care Medicine, Affiliated Hospital of Hebei University, Baoding, China

**Keywords:** gut microbes, intestinal microbiota, meta-analysis, pancreatic cancer, systematic review

## Abstract

**Background:**

Pancreatic cancer (PC) is a highly aggressive malignancy with an asymptomatic early stage, often resulting in rapid invasion of adjacent tissues and organs and a high mortality rate. Recent research has increasingly examined the gut microbiome as a potential factor in PC pathogenesis. Although changes in gut microbiota composition have been reported in patients with PC, a comprehensive evaluation of the relationship between gut microbiota and PC has not been systematically conducted.

**Methods:**

A systematic search of PubMed, Web of Science, Embase, and Cochrane databases was conducted, and a meta-analysis was performed on 10 studies including a total of 324 patients with PC.

**Results:**

The meta-analysis did not identify a statistically significant difference in *α*-diversity and microbial richness between patients with PC and those in the control group. However, a decrease in *Bacteroides*, *Neisseria*, *Porphyromonas*, *Prevotella*, and *Actinomyces* was observed in patients with PC, while *Fusobacterium*, *Rothia*, *Streptococcus*, *Veillonella*, and *Escherichia-Shigella* levels were increased.

**Conclusion:**

This meta-analysis demonstrates an association between PC and changes in gut microbiota composition at both the species and genus levels.

**Systematic review registration:**

https://www.crd.york.ac.uk/PROSPERO/recorddashboard, identifier CRD42023468159.

## 1 Introduction

Pancreatic cancer (PC) is among the most aggressive malignant neoplasms, characterized by an insidious onset and poor prognosis (Siegel et al., 2018). Approximately 10% of patients diagnosed with PC have a familial predisposition (Klein, 2012; Klein et al., 2004). The high mortality rate associated with PC is primarily attributed to delayed diagnosis and limited therapeutic options (Kamisawa et al., 2016). The disease often presents with nonspecific symptoms that become apparent only at advanced stages, frequently rendering the tumor unresectable or indicative of metastatic progression, thereby limiting therapeutic efficacy. Epidemiological data indicate an estimated annual incidence of approximately 216,000 new cases worldwide. The 1-year survival rate is approximately 23%, while the 5-year survival rate remains as low as 5% (McGuire, 2016). Currently, the primary strategies for PC prevention and early detection rely on advanced pancreatic imaging to identify precancerous lesions (Siegel et al., 2022; Catalano et al., 2003; Ichikawa et al., 2001). However, due to the impracticality of large-scale population screening with this approach, there is a critical need to develop novel diagnostic techniques to improve early detection, prognostication, and overall survival outcomes.

Recent studies have proposed a potential association between the intestinal microbiota and various gastrointestinal malignancies, including PC (Zitvogel et al., 2018; Gopalakrishnan et al., 2018; Sepich-Poore et al., 2021). A study by Chen et al. (2023) reported substantial changes in microbial community composition in patients with PC compared to control groups. An increased abundance of oral pathogenic bacteria (*Granulicatella*, *Peptostreptococcus*, *Alloprevotella*, *Veillonella*, *Solobacterium*, *Streptococcus*, etc.) was observed in patients with PC. Within the intestinal microbiota, beneficial bacteria such as *Bifidobacterium* and *Butyricococcus*, including probiotics, demonstrated a significant reduction, whereas opportunistic bacteria (*Prevotella*, *Escherichia Shigella*, *Peptostreptococcus*, *Actinomyces*, etc.) were significantly increased. Further studies by Tan et al. (2022) identified a relationship between *Porphyromonas gingivalis*, a pathogen implicated in chronic periodontitis, and the development of pancreatic ductal adenocarcinoma (PDAC) (Wei et al., 2019). Mechanistically, intratumoral *P. gingivalis* promotes PC progression by increasing the secretion of neutrophilic chemokines and neutrophil elastase (NE). The potential role of *Helicobacter pylori* in PDAC development is under investigation (Kumar et al., 2020; Hirabayashi et al., 2019; Nilsson et al., 2006). However, its classification as a definitive risk factor for PC remains controversial.

The introduction of 16S rRNA sequencing technology and fluorescence *in situ* hybridization (FISH) has facilitated the identification of significant changes in the abundance and composition of intestinal microbiota in patients with PC compared to healthy controls. A study by Kartal et al. (2022) identified distinct associations between various microbial groups present in intestinal and pancreatic tissues. The enrichment of specific bacterial taxa in tumor tissue compared to adjacent healthy tissue indicates a potential link between PC and intestinal microbiota. Similarly, findings by Geller et al. (2017) demonstrated an increased presence of bacterial DNA in PC tissue compared to normal pancreatic tissue. Additionally, Aykut et al. (2019) utilized FISH technology to detect bacterial ribosomal DNA in 76% of patients with PC, whereas bacterial ribosomal DNA was observed in only 15% of patients with normal pancreatic tissue.

Although previous studies have reported alterations in the gut microbiota composition of patients with PC, a comprehensive systematic evaluation of the relationship between gut microbiota and PC is still lacking. This study, through a meta-analysis, reveals an association between PC and changes in gut microbiota composition. It aims to provide a comprehensive clinical reference to improve the diagnosis and treatment of PC.

## 2 Materials and methods

### 2.1 Search strategy

A comprehensive search was conducted across four electronic databases (PubMed, EMBASE, Cochrane Central Register of Controlled Trials, and Web of Science) from their inception to July 2023. The search strategy was systematically developed using the PICOS tool: (P) Population—patients diagnosed with PC; (C) Comparator—healthy control group; and (O) Outcomes—distribution characteristics of gut microbiota in patients with PC. Detailed data regarding the search strategy is provided in Table 1.

**Table 1:** Search strategy on PubMed.

Step	Search Strategy
#1	((Pancreatic Neoplasms[MeSH Major Topic]) OR (Pancreatic Carcinoma[MeSH Major Topic])) OR (Pancreatic cancer[MeSH Major Topic])
#2	((((((((((((((((((((Pancreatic Neoplasms[Title/Abstract]) OR (Pancreatic Carcinoma[Title/Abstract])) OR (Pancreatic cancer[Title/Abstract])) OR (Neoplasm, Pancreatic[Title/Abstract])) OR (Pancreatic Neoplasm[Title/Abstract])) OR (Pancreas Neoplasms[Title/Abstract])) OR (Neoplasm, Pancreas[Title/Abstract])) OR (Neoplasms, Pancreas[Title/Abstract])) OR (Pancreas Neoplasm[Title/Abstract])) OR (Neoplasms, Pancreatic[Title/Abstract])) OR (Cancer of Pancreas[Title/Abstract])) OR (Pancreas Cancers[Title/Abstract])) OR (Pancreas Cancer[Title/Abstract])) OR (Cancer, Pancreas[Title/Abstract])) OR (Cancers, Pancreas[Title/Abstract])) OR (Pancreatic Cancer[Title/Abstract])) OR (Cancer, Pancreatic[Title/Abstract])) OR (Cancers, Pancreatic[Title/Abstract])) OR (Pancreatic Cancers[Title/Abstract])) OR (Cancer of the Pancreas[Title/Abstract])) OR (Familial Pancreatic carcinoma[Title/Abstract])
#3	#1 OR #2
#4	Gastrointestinal Microbiome[MeSH Major Topic]
#5	(((((((((((((((((((((((((((((((((Gastrointestinal Microbiome[Title/Abstract]) OR (Gastrointestinal Microbiomes[Title/Abstract])) OR (Microbiome, Gastrointestinal[Title/Abstract])) OR (Gut Microbiome[Title/Abstract])) OR (Gut Microbiomes[Title/Abstract])) OR (Microbiome, Gut[Title/Abstract])) OR (Gut Microflora[Title/Abstract])) OR (Microflora, Gut[Title/Abstract])) OR (Gut Microbiota[Title/Abstract])) OR (Gut Microbiotas[Title/Abstract])) OR (Microbiota, Gut[Title/Abstract])) OR (Gastrointestinal Flora[Title/Abstract])) OR (Gastrointestinal Microbiota[Title/Abstract])) OR (Gastrointestinal Microbiotas[Title/Abstract])) OR (Microbiota, Gastrointestinal[Title/Abstract])) OR (Gastrointestinal Microbial Community[Title/Abstract])) OR (Gastrointestinal Microbial Communities[Title/Abstract])) OR (Microbial Community, Gastrointestinal[Title/Abstract])) OR (Gastrointestinal Microflora[Title/Abstract])) OR (Microflora, Gastrointestinal[Title/Abstract])) OR (Gastric Microbiome[Title/Abstract])) OR (Gastric Microbiomes[Title/Abstract])) OR (Microbiome, Gastric[Title/Abstract])) OR (Intestinal Microbiome[Title/Abstract])) OR (Intestinal Microbiomes[Title/Abstract])) OR (Microbiome, Intestinal[Title/Abstract])) OR (Intestinal Microbiota[Title/Abstract])) OR (Intestinal Microbiotas[Title/Abstract])) OR (Microbiota, Intestinal[Title/Abstract])) OR (Intestinal Microflora[Title/Abstract])) OR (Microflora, Intestinal[Title/Abstract])) OR (Intestinal Flora[Title/Abstract])) OR (Enteric Bacteria[Title/Abstract])) OR (Bacteria, Enteric[Title/Abstract])
#6	#4 OR #5
#7	#3 AND #6

### 2.2 Inclusion criteria

(1) Inclusion of an experimental group consisting of patients diagnosed with PC. (2) Inclusion of a healthy control group. (3) Studies reporting relevant outcomes, specifically addressing gut microbiota composition and microbial diversity, were considered in the review.

### 2.3 Exclusion criteria

(1) Studies with incomplete or unreported data were excluded. (2) Studies originating from non-randomized controlled trials, including quasi-randomized controlled trials, animal studies, study protocols, conference abstracts, case reports, or correspondence, were not considered.

### 2.4 Study selection

The literature was screened and excluded using the EndNote reference management software. Initially, a preliminary screening of literature titles was conducted by two researchers to remove duplicates, non-randomized controlled trials, review articles, conference papers, study protocols, and correspondence. Subsequently, abstracts of the identified studies were independently reviewed by both researchers to determine eligibility for inclusion or exclusion. The remaining studies underwent a comprehensive evaluation to identify those meeting the inclusion criteria. Throughout the screening process, both researchers conducted independent assessments, and any discrepancies were resolved through mutual comparison. In cases where disagreements persisted, a third researcher was consulted to reach a consensus.

### 2.5 Data extraction

A standardized, pre-determined nine-item data extraction form was used to systematically collect and record data for inclusion in the study. The extracted data were categorized under the following headings: (1) author, (2) year of publication, (3) country, (4) study population, (5) sample size, (6) mean age, (7) microbial alpha diversity, (8) bacterial species, and (9) bacterial genus.

### 2.6 Risk of bias quality assessment

The quality of the selected case-control studies was evaluated using the Newcastle–Ottawa Scale, which consists of eight items assessing three key dimensions: selection, comparability, and exposure. The total score ranges from 6 to 9, with higher scores indicating a more rigorous quality assessment.

### 2.7 Data analysis

A meta-analysis was conducted when sufficient trial data reporting the same outcome was accumulated. Continuous data were analyzed using the mean difference (MD) and 95% confidence intervals (CIs). The standardized mean difference (SMD) was utilized for analysis due to variations in measurement units across the included studies. Meta-analysis results were presented using forest plots, and statistical significance for the overall effect was determined at *p* < 0.05. Heterogeneity was assessed using the *I*^2^ statistic. A fixed-effects model was used if *I*^2^ ≤ 50%, indicating low heterogeneity among studies. If *I*^2^ > 50%, indicating substantial heterogeneity, a sensitivity analysis was performed to find the source. If the source remained unexplained, a random-effects model was applied.

## 3 Results

### 3.1 Study and identification and selection

A total of 2,237 documents were retrieved from the electronic database. After the removal of duplicate entries, 1,866 documents remained for title and abstract screening, resulting in the exclusion of 1,771 documents. A comprehensive review was then conducted on the remaining 95 papers, of which 26 were partially examined and subsequently excluded. The final 69 documents were excluded due to factors such as non-randomized controlled trials, incomplete data, inclusion of conference papers or animal studies, and failure to meet the predetermined inclusion criteria. Ultimately, 10 documents met the inclusion criteria and were incorporated into this study.

### 3.2 Quality assessment of the included studies

The Newcastle–Ottawa Scale (NOS) scores for each study across the three domains (Selection, Comparability, and Exposure) were showed in Supplementary Table 1. Two studies were categorized as low-quality literature, six were classified as moderate quality, and two were designated as high quality. One of the low-quality studies was classified as such due to significant discrepancies in sample variables between the case and control groups. The other low-quality study used pancreatitis as the control group, introducing multiple confounding factors that may have contributed to potential bias in the final results.

### 3.3 Characteristics of the selected observational trials

**Figure 1:**
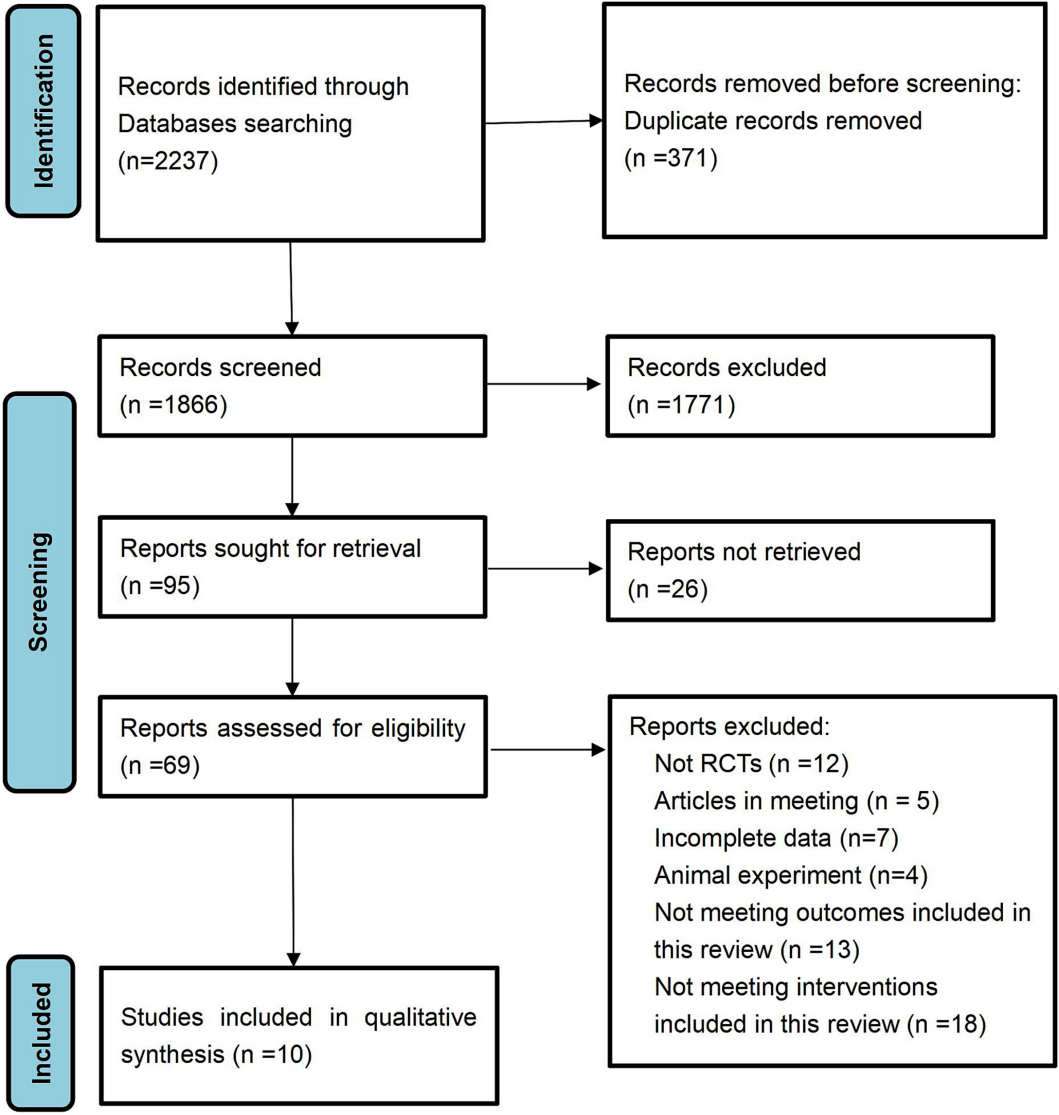
Flow diagram of literature selection.

Ten studies were included in the analysis (Figure 1). The meta-analysis encompassed a study population consisting of 324 patients diagnosed with PC and 413 age-matched healthy control participants (Table 2). Geographically, seven studies (70%) were conducted in Asian regions, including China, Japan, South Korea, and Israel, while the remaining three studies (30%) were conducted in western populations, specifically in the United States and Germany. The assessment involved a comparative analysis of species diversity and richness among the included bacterial species. All 10 studies accounted for confounding factors related to gender. Fecal samples were collected for sequencing, with 16S ribosomal RNA sequencing being the predominant methodology (9 out of 10 studies, 90%), while one study (10%) utilized shotgun metagenomics.

**Table 2:** Characteristics of the studies included in the meta-analysis.

Author	Country	Year	Population	Age (mean ± SD)	Total/male/female	Diversity	Phyium	Genus
Shiro Kohi	USA	2022	PC&HC	Adults	PC: 74 HC: 134	Shannon OTUS	Bacteroidetes Firmicutes Proteobacteria Actinobacteria Fusobacteria	Veillonella Streptococcus Prevotella Fusobacterium Escherichia-Shigella Porphyromonas Rothia Neisseria Actinomyces
Ece Kartal	Germany	2022	PC&HC	Adults	PC: 45 HC: 43	Shannon Simpson		AKKermansia Veillonella Streptococcus
Tian Chen	China	2023	PC&HC	PC: 56.58 (9.71) HC: 56.35 (6.90)	PC: 40 HC: 39/20/19	Chao-1 OTUS	Bacteroidetes Firmicutes Proteobacteria Actinobacteria Fusobacteria	AKKermansia Veillonella Streptococcus Prevotella Fusobacterium Escherichia-Shigella Porphyromonas Rothia Neisseria Actinomyces Bacteroides
Senju Hashimoto	Japan	2023	PC&HC	PC: 73 (4.75) HC: 54 (4.81)	PC: 5/2/3 HC: 68/29/39	Shannon		Veillonella Streptococcus Actinomyces
Pedro J. Torres	USA	2015	PC&HC	Adults	PC: 8/6/2 HC: 22/12/10	Chao-1 OTUS	Bacteroidetes Firmicutes Proteobacteria Actinobacteria Fusobacteria	Streptococcus Fusobacterium Porphyromonas Neisseria
Qi-Xiang Mei	China	2018	PC&HC	PC: 56.8 (5.1) HC: 55.4 (6.2)	PC: 14/9/5 HC: 14/9/5	Shannon Chao-1 OTUS Simpson	Bacteroidetes Firmicutes Proteobacteria	Escherichia-Shigella Porphyromonas
Jin-Yong Jeong	Korea	2020	PC&HC	PC: 65.0 (8.2) HC: 65.0 (8.2)	PC: 15/4/11 HC: 15/4/11	Chao-1 Shannon ACE Simpson		AKKermansia Streptococcus Prevotella
Elizabeth Half	Israel	2019	PC&HC	PC: 68.9 (6.2) HC: 59 (8.7)	PC: 30/16/14 HC: 13/6/7	Shannon	Bacteroidetes	AKKermansia Bacteroides
Zhigang Ren	China	2017	PC&HC	PC: 56 (11.25) HC: 52 (8)	PC: 85/47/38 HC: 57/36/21	Shannon Simpson Chao-1	Bacteroidetes Firmicutes Proteobacteria	Veillonella Prevotella
Xue-Yuan Wang	China	2022	PC&HC	PC: 63.13 (13.11) HC: 61.13 (12.14)	PC: 8/6/3 HC: 8/5/3	Chao-1 Shannon ACE Simpson	Bacteroidetes Firmicutes Proteobacteria Actinobacteria Fusobacteria	

### 3.4 Alpha diversity

**Figure 2:**
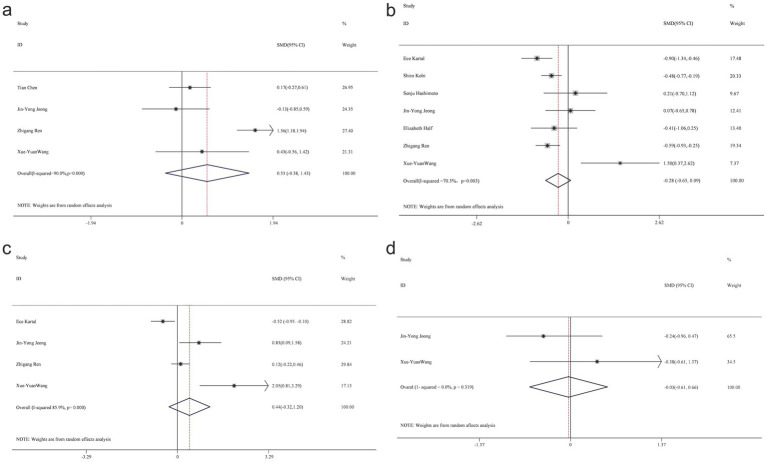
Forest map of alpha diversity differences by Chao index **(a)** Shannon index **(b)** Simpson **(c)** and ACE **(d)**.

Alpha diversity is a metric that reflects the richness and variety of microbial communities within a given sample. The analysis focused on alpha diversity indexes such as Chao1, Shannon, Simpson, and ACE, to compare patients with PC and healthy controls (Figure 2). The Chao1 index, a relatively novel parameter in PC research, did not exhibit a statistically significant difference between patients with PC (*n* = 85) and the control group (*n* = 98) across five studies, with a combined standardized mean difference (SMD) of 0.53 (95% CI, −0.38 to 1.43) and high heterogeneity, particularly evident in the Shannon index.

Eight studies, which included patient samples (*n* = 276) and control groups (*n* = 352), reported findings on the Shannon index, with combined estimates indicating no significant intergroup differences (SMD = −0.28; 95% CI, −0.65 to 0.09), while demonstrating high heterogeneity, as shown in the Shannon diagram. Four studies contributed data on the Simpson index (*n* = 82 patients; *n* = 80 controls), revealing no statistically significant difference between the two groups (SMD = 0.44; 95% CI, −0.32 to 1.2), with considerable heterogeneity (85.9%).

A heterogeneity test was conducted, and a random-effects model was applied due to the observed heterogeneity in diversity indices. The respective data for the Chao1 index (*t*-value = −0.9, *p* = 0.463), Shannon index (*t*-value = 2.45, *p* = 0.058), and Simpson’s index (*t*-value = 1.59, *p* = 0.254) indicated no significant differences between the PC and HC groups.

### 3.5 Summary of representative groups

A systematic compilation of representative taxonomic groups identified in patients with PC and healthy controls was conducted at both the species and genus levels.

**Figure 3:**
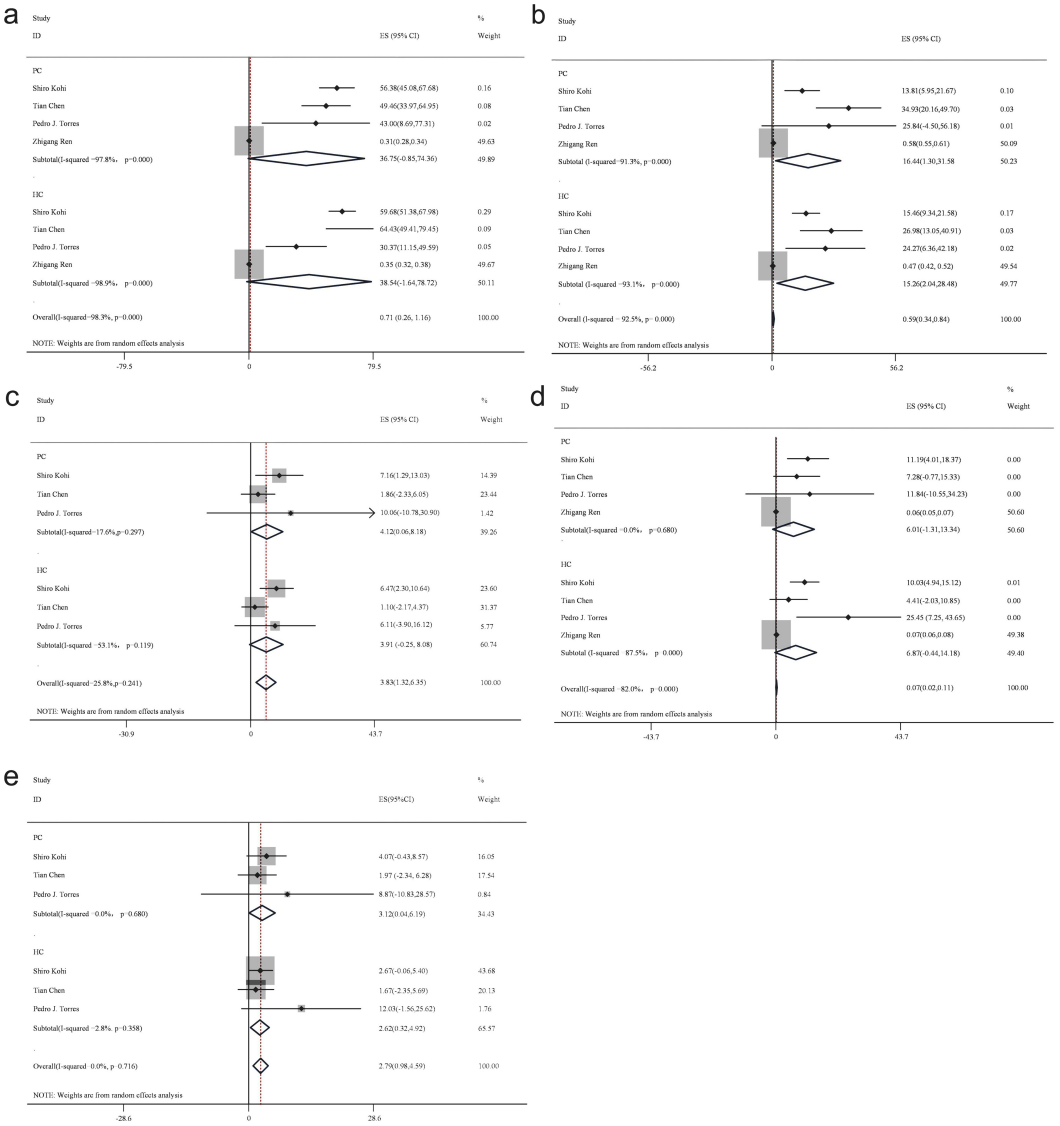
Forest plot of phylum level gut bacteria. Firmicutes **(a)**, Bacteroidetes **(b)**, Actinobacteria **(c)**, Proteobacteria **(d)**, and Fusobacteria **(e)**.

At the phylum level, data from 10 studies identified five bacterial phyla: Firmicutes, Bacteroidetes, Actinobacteria, Proteobacteria, and Fusobacteria. However, the findings for each taxonomic unit exhibited inconsistencies (Figure 3).

Firmicutes, a dominant phylum within the human gut microbiota, demonstrated a reduced abundance in patients with PC compared to the healthy control group. The proportion of Firmicutes in PC group samples was estimated at 36.8% (95% CI: −0.85 to 74.36), while in the healthy control group, it was 38.5% (95% CI: −1.64 to 78.72), yielding a bacterial percentage of 0.96 across both groups using a random-effects model.

In contrast, Bacteroidetes exhibited an increased presence in patients with PC compared to the healthy control group. The random-effects model estimated the proportion of Bacteroidetes in PC group samples at 16.4% (95% CI: 1.3 to 31.58), while in healthy control samples, it was 15.3% (95% CI: 2.04 to 28.48), resulting in a bacterial percentage of 1.07.

Proteobacteria demonstrated a lower abundance in patients with PC compared to the healthy control group. The random-effects model estimated the proportion of Proteobacteria in PC group samples at 6% (95% CI: −1.31 to 13.34), whereas in the healthy control group, it was 6.9% (95% CI: −0.44 to 14.18), yielding a bacterial percentage of 0.87 across both groups.

Conversely, Actinobacteria revealed an increased abundance in patients with PC compared to the healthy control group. The random-effects model estimated the proportion of Actinobacteria in PC group samples at 4.1% (95% CI: 0.06 to 8.18), while in healthy control samples, it was 3.9% (95% CI: −0.25 to 8.08), resulting in a bacterial percentage of 1.05 across both groups.

**Figure 4:**
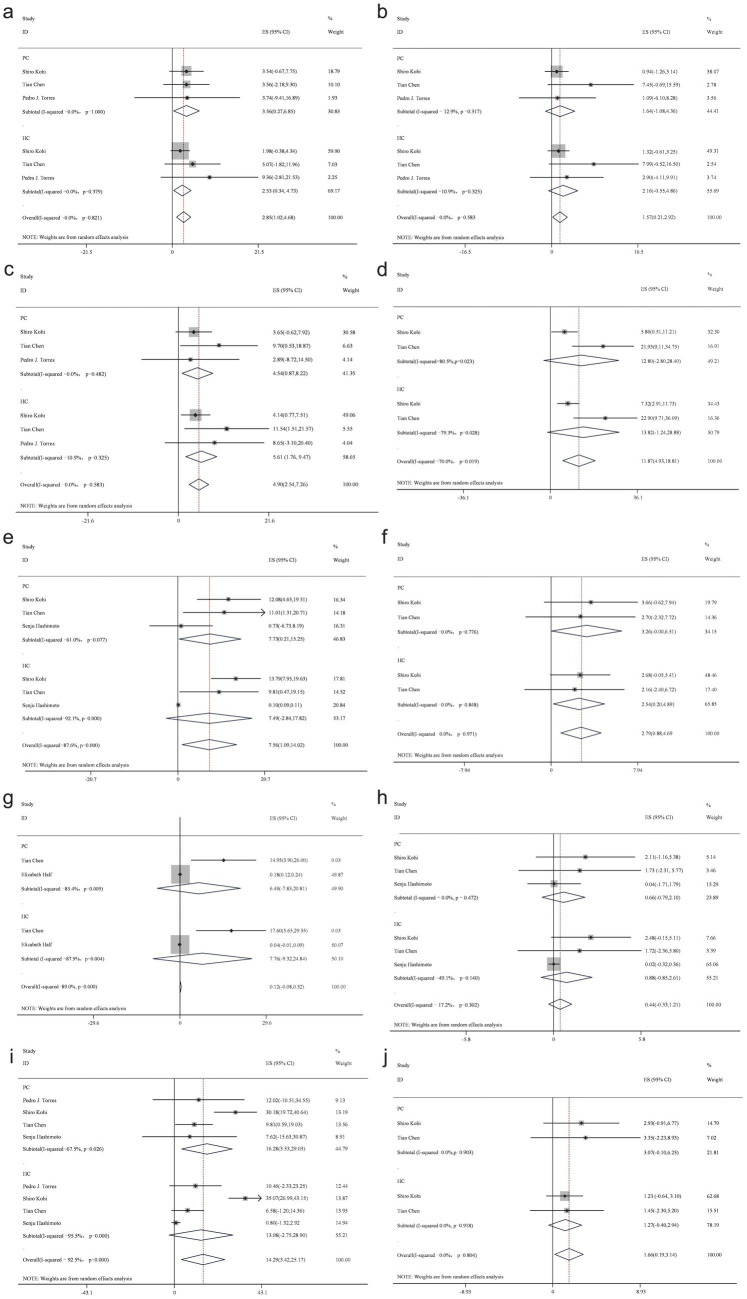
Forest plot of genus level gut bacteria. *Fusobacterium* **(a)**, *Neisseria* **(b)**, *Porphyromonas* **(c)**, *Prevotella* **(d)**, *Rothia* **(e)**, *Streptococcus* **(f)**, *Veillonella* **(g)**, *Actinomyces* **(h)**, *Veillonella* **(i)**, and *Escherichia-Shigella* **(j)**.

At the genus level, an analysis of 10 studies identified 11 bacterial genera. Findings from more than three studies consistently demonstrated a decreased abundance of *Bacteroides*, *Neisseria*, *Porphyromonas*, *Prevotella*, and *Actinomyces* in patients with PC, while *Fusobacterium*, *Rothia*, *Streptococcus*, *Veillonella*, and *Escherichia*-*Shigella* were more abundant compared to the healthy control group (Figure 4).

### 3.6 Publication bias test

Distinct funnel plots were generated for each outcome indicator to evaluate the potential presence of publication bias (Lin and Chu, 2018). Egger’s test and Begg’s test were used to quantitatively evaluate the symmetry of funnel plots (Supplementary Table 2). The results suggest that there may be a small sample effect or publication bias among the studies.

## 4 Discussion

This study constitutes the first meta-analysis on intestinal microbiota in patients with PC, based on the current knowledge levels. A comprehensive review of 2,237 articles was conducted, leading to the rigorous selection of 10 case-control studies, encompassing 324 patients with PC and 413 age-matched healthy controls.

Notable differences were identified at both the species and genus taxonomic levels upon detailed evaluation. At the phylum level, an increased abundance of Bacteroides, Fusobacterium, and Actinobacteria was observed in patients with PC compared to the healthy control group, along with a reduction in Firmicutes and Proteobacteria.

Further analysis at the genus level revealed distinct variations in microbial composition within fecal samples from patients with PC compared to healthy controls. A decreased presence of *Bacteroides*, *Neisseria*, *Porphyromonas*, *Prevotella*, and *Actinomyces* was noted, while an increased abundance of *Fusobacterium*, *Rothia*, *Streptococcus*, *Veillonella*, and *Escherichia-Shigella* was observed.

These findings highlight the potential of microbial markers as non-invasive tools for PC diagnosis, early detection, and therapeutic strategies. The identification of such microbiota-associated signatures contributes to the efforts aimed at reducing PC-related mortality. The clinical significance of this study lies in its contribution to advancing knowledge in the field and informing future research on microbiota-based approaches in PC management.

The assessment of alpha diversity is widely used in scientific research (Lozupone and Knight, 2008). It is generally hypothesized that greater microbial diversity is beneficial to the host, and consequently, a reduction in alpha diversity is expected in patients with PC. This assumption is supported by a study conducted by Sidiropoulos et al. (2024), which analyzed the fecal microbiomes of patients with PC. They found that the Chao1 and Shannon indices of PC patients were significantly decreased.

However, the meta-analysis did not identify statistically significant differences in diversity indices between the two survivor groups, providing a nuanced perspective on the proposed reduction in alpha diversity among patients with PC.

An increased abundance of *Bacteroides* and *Fusobacterium* was observed in patients with PC compared to healthy controls. This finding is consistent with an investigation by Kohi et al. (2022) which reported an enrichment of *Fusobacterium* in the duodenal fluid microbiota of patients with PDAC, particularly those with shorter survival durations compared to the healthy control group. *Fusobacterium* has been identified as an independent adverse prognostic biomarker for PC (Dapito et al., 2012).

Further exploration into the carcinogenic role of *Fusobacterium* was conducted by Klein (2012) who delineated its interaction with E-cadherin in epithelial cells. This interaction promotes phosphorylation and internalization of extracellular vesicles on the membrane, leading to the activation of the Wnt pathway. As a result, reduced phosphorylation of catenin occurs, facilitating β-catenin accumulation in the cytoplasm and its subsequent translocation to the nucleus (Bangolo et al., 2023). The enhancement of Wnt signaling and activation of the Wnt/β-catenin pathway have been identified as key mechanisms in the initiation of PC. Recent studies have shown that certain species of *Fusobacterium*, such as *Fusobacterium nucleatum*, can stimulate the production of specific cytokines like GM-CSF and CXCL1 (Tabrizi et al., 2024). GM-CSF promotes the proliferation, growth, and spread of pancreatic cancer cells in the body. CXCL1, a chemokine closely related to inflammation and immune responses, plays a key role in promoting pancreatic cancer cell metastasis and conferring chemoresistance (Udayasuryan et al., 2022).

At the genus level, *Prevotella* was identified as the predominant genus within the intestinal microbiota. Recent studies have convincingly demonstrated that *Prevotella* is involved in the development of PC, although the precise mechanisms remain unclear (Chen et al., 2023; Eid et al., 2024). Evidence suggests that *Prevotella* participates in the pathogenesis of rheumatoid arthritis in murine models via activation of the Th17/IL-17 pathway. Additionally, *Prevotella* accelerates the progression of pancreatic intraepithelial neoplasia (Maeda et al., 2016; Bernard, 2014; Alpizar-Rodriguez et al., 2019; McAllister et al., 2014). These findings indicate the possibility that *Prevotella* contributes to PC development through activation of the Th17/IL-17 pathway.

Furthermore, the meta-analysis revealed an increased abundance of *Streptococcus* and *Veillonella* in individuals with PC, which is consistent with findings from two independent group studies conducted in China and Israel. These studies reported a similar enrichment of *Streptococcus* and *Veillonella* within the intestinal microbiota of patients with PC, indicating potential microbial commonalities associated with PC across different ethnic groups (Ren et al., 2017).

The meta analysis of this study systematically revealed gut microbiota characteristics of PC patients, finding that changes in *Fusobacterium* and *Streptococcus* might link to PC progression. Although clinical information gaps (e.g., staging, treatment status) in the original data restricted the analysis, the results still offer key clues for developing non-invasive microbiota-based diagnostic tools and targeted interventions.

Future research should focus on the following directions: First, prospective cohort or multicenter studies are needed to clarify the dynamic changes in the gut microbiota across different tumor stages (such as early vs. late stages), treatment statuses (pre-/post-surgery, chemotherapy/radiotherapy), and comorbidities (diabetes, obesity, etc.), in order to identify biomarkers with clinical translation value. Second, the integration of metagenomic and metabolomic approaches can help elucidate the molecular mechanisms of specific strains’ functional genes and their metabolic products (such as short-chain fatty acids, bile acid derivatives) in PC development, offering a theoretical basis for targeted interventions. Finally, it is recommended to establish unified clinical data reporting standards (such as the STROBE guidelines) to ensure the standardized recording of variables like tumor stage, treatment details, and comorbidities. This will enhance the comparability and integrability of data across studies. These explorations will help bridge the gap from “correlation” to “causality” and ultimately enable precision diagnosis and treatment strategies based on microbiome regulation.

## 5 Strengths and limitations

This study adhered strictly to predefined inclusion and exclusion criteria, incorporating a comprehensive search, screening, and systematic evaluation in accordance with the Preferred Reporting Items for Systematic Reviews and Meta-Analyses (PRISMA) guidelines (Page et al., 2021a,b). A total of 10 studies involving 324 patients with PC were included in the analysis, with results derived through an objective analytical approach.

Despite these methodological rigors, several limitations inherent to this meta-analysis should be acknowledged: (1) The body of available literature and research suitable for inclusion remained limited. (2) Substantial variability in sample sizes across included studies may disproportionately amplify effects from underpowered trials. (3) Some indicators, such as alpha diversity, show a high degree of heterogeneity. (4) Geographical differences: Although efforts were made to minimize heterogeneity by controlling for variables such as gender, race, diet, sample size, and geographic distribution, there were unavoidable discrepancies which persisted across studies. (5) Methodological differences: In analyses utilizing 16S rRNA sequencing technology, potential sources of variability—including differences in researchers, sequencing timelines, sequencing platforms, and experimental methodologies—may have contributed to inconsistencies in findings. (6) The pathophysiological progression of PC itself may influence changes in the intestinal microbiota, further complicating interpretations of microbiota changes. (7) This study identifies an association between intestinal microbiota composition and PC but does not establish a causal relationship.

## 6 Conclusion

The findings of this meta-analysis indicate no statistically significant differences in the alpha diversity index between patients with PC and healthy controls. However, an association was observed between the increased abundance of specific bacteria—*Fusobacterium*, *Veillonella*, and *Streptococcus*—and PC. It is important to emphasize that this meta-analysis establishes only a correlation between intestinal microbiota composition and the pathogenesis of PC, with conclusions constrained by the limited body of available research. Therefore, further primary and clinical studies are necessary to elucidate the complex interplay between intestinal microbiota dysbiosis and the pathogenesis and progression of PC.
