# Supplementary material for: Changes in intestinal microbiota in patients with pancreatic cancer: a systematic review and meta-analysis
Source: Front Microbiol. 2025 Sep 1;16:1619323. doi: 10.3389/fmicb.2025.1619323 (PMC12434105; doi:10.3389/fmicb.2025.1619323)
Supplement: Supplementary file 1 [file Table_1.DOCX]

Supplementary Table 1. The Newcastle-Ottawa Scale (NOS) scores for each study

| Author | selection | comparability | exposure |
| --- | --- | --- | --- |
| Tian Chen | 4 | 1 | 3 |
| Ece Kartal | 4 | 2 | 3 |
| Senju Hashimoto | 3 | 1 | 3 |
| Zhigang Ren | 4 | 1 | 3 |
| Xue-YuanWang | 4 | 2 | 3 |
| Elizabeth Half | 4 | 2 | 3 |
| Shiro Kohi | 3 | 2 | 3 |
| Pedro J. Torres | 4 | 1 | 3 |
| Qi-Xiang Mei | 4 | 2 | 3 |
| Jin-Yong Jeong | 4 | 1 | 3 |
