# Supplementary material for: Changes in intestinal microbiota in patients with pancreatic cancer: a systematic review and meta-analysis
Source: Front Microbiol. 2025 Sep 1;16:1619323. doi: 10.3389/fmicb.2025.1619323 (PMC12434105; doi:10.3389/fmicb.2025.1619323)
Supplement: Supplementary file 2 [file Table_2.DOCX]

Supplementary Table 2. The results of Egger's Test and Begg's Test

| Name | *P* value of Egger's Test | *P* value of Begg's Test |
| --- | --- | --- |
| Chao-1 | 0.463 | 1 |
| ACE | - | 1 |
| Shannon | 0.058 | 0.072 |
| Simpson | 0.254 | 0.308 |
| *Bacteroidetes* | 0 | 0.631 |
| *Firmicutes* | 0 | 1 |
| *Fusobacteria* | 0 | 0.14 |
| *Proteobacteria* | 0 | 0.631 |
| *Actinobacteria* | 0.1 | 0.46 |
| *Veillonella* | 0 | 1 |
| *Streptococcus* | 0.2 | 0.337 |
| *Prevotella* | 1 | 0.157 |
| *Fusobacterium* | 0 | 0.027 |
| *Escherichia-Shigella* | - | 0.157 |
| *Porphyromonas* | 0 | 0.027 |
| *Rothia* | - | 0.157 |
| *Neisseria* | 0 | 0.46 |
| *Actinomyces* | 0 | 0.46 |
| *Bacteroides* | - | 0.157 |
